# Densification-Induced Structure Changes in Basolite MOFs: Effect on Low-Pressure CH_4_ Adsorption

**DOI:** 10.3390/nano10061089

**Published:** 2020-06-01

**Authors:** David Ursueguía, Eva Díaz, Salvador Ordóñez

**Affiliations:** Catalysis, Reactors and Control Research Group (CRC), Department of Chemical and Environmental Engineering, University of Oviedo, 33006-Oviedo, Spain; ursueguiadavid@uniovi.es (D.U.); diazfeva@uniovi.es (E.D.)

**Keywords:** coordination polymers, methane storage, XRD crystallinity measurements, mechanical shaping, compaction, VAM, gas separation, MOF pelletization

## Abstract

Metal-organic frameworks’ (MOFs) adsorption potential is significantly reduced by turning the original powder into pellets or granules, a mandatory step for their use at industrial scale. Pelletization is commonly performed by mechanical compression, which often induces the amorphization or pressure-induced phase transformations. The objective of this work is the rigorous study of the impact of mechanical pressure (55.9, 111.8 and 186.3 MPa) onto three commercial materials (Basolite C300, F300 and A100). Phase transformations were determined by powder X-ray diffraction analysis, whereas morphological changes were followed by nitrogen physisorption. Methane adsorption was studied in an atmospheric fixed bed. Significant crystallinity losses were observed, even at low applied pressures (up to 69.9% for Basolite C300), whereas a structural change occurred to Basolite A100 from orthorhombic to monoclinic phases, with a high cell volume reduction (13.7%). Consequently, adsorption capacities for both methane and nitrogen were largely reduced (up to 53.6% for Basolite C300), being related to morphological changes (surface area losses). Likewise, the high concentration of metallic active centers (Basolite C300), the structural breathing (Basolite A100) and the mesopore-induced formation (Basolite F300) smooth the dramatic loss of capacity of these materials.

## 1. Introduction

Energy demand estimations for the next decades, mainly due to the global population and industrialization process increments, boost the development of techniques and processes able to make the most of available resources [1]. What is more, the recent COVID-19 pandemic, with millions of people confined to their homes, pointed out even more our domestic reliance on electricity. In most economies that have taken strong confinement measures in response to the coronavirus, electricity demand has declined by around 15%, and the share of variable renewables like wind and solar had become higher than normal [2]. Even when electricity from wind and solar would satisfy the majority of demand, systems need to maintain flexibility in order to be able to ramp up other sources of generation quickly when the pattern of supply shifts, such as when the sun sets. That is, electricity system operators have to constantly balance demand and supply in real time to prevent blackouts, which in recent times occurred mainly during periods of low demand [2]. In this context, natural gas power plants can quickly ramp generation up or down at short notice, providing in this way flexibility, underlining the critical role of gas in the longed-for clean energy transition.

In the natural gas industry, methane purification is a major process for upgrading the streams [3]. In these streams, methane concentration is originally elevated (>90%), so satisfactory results have been reported using fixed-bed adsorption techniques [4,5]. In these cases, the usual practice is to separate the component that is in lower concentration by adsorption (typically CO_2_). Adsorbents usually used for this purpose are activated carbons and zeolites, which have good CO_2_ adsorption yields and their cost is relatively low [6,7]. On the other hand, these techniques present difficulties when methane is the component with the lowest concentration in the stream. Activated carbons and zeolites present low selectivity towards methane with respect to other very similar compounds in molecular size and polarity, like nitrogen [8,9]. This is the case of one of the new alternative methane sources that has begun to be studied in recent years, the recovery of methane from ventilation gases from mining exploitation (VAM). Until now, these streams, which contain typically less than 1% in methane, had been burned directly, with the need of an auxiliary fuel. VAM could be used in order to obtain energy or chemical products, as well as to prevent greenhouse gas emissions into the atmosphere [10,11]. For these operations to be profitable, it is necessary to perform a previous concentration step, whose success depends on the separation capacity of the adsorbent used [12].

Among the materials studied for this purpose, due to its amazing properties, metal-organic frameworks (MOFs) have been shown to present large adsorption and gas separation yields [13,14], these being among the most promising materials in this field. Their high specific surface area (even values up to 6255 m^2^/g [15]) combined with high total pore volume (1.303 cm^3^/g [16]) and great porosity (91.1% [17]) are responsible for the large adsorption capabilities, exceeding in the majority of cases other common materials [18]. The materials’ structure is made up by an organic ligand, such as imidazole or pyrazine, which links different metal ions or clusters corresponding to each MOF type (copper, aluminium, etc.). These combinations form a cage-like structure that is repeated continuously, conferring on these materials a high degree of crystallinity [19]. Two of the main characteristics of the MOFs are the flexibility in the design, which means a huge variety of organic ligands and metallic ions that allow on-demand materials to be made, and the pore functionalization, presenting high interesting adsorptive and catalytic properties. The possibility of performing a large number of combinations has led to an astonishing number of works related to the synthesis of MOFs suitable for different applications, which include gas storage and separation [20]. For example, in the case of methane separation from other gases, Arami-Niya et al. [21] have tested the zeolitic imidazolate framework (ZIF-7) for the separation of methane from nitrogen, obtaining a selectivity of more than 10 for an equimolar mixture at 303 K. In addition, other authors such as Eyer et al. [22] have studied different materials capable of selectively adsorbing methane from air mixtures, obtaining promising results in the case of HKUST-1, with a selectivity methane/nitrogen of 2.8 and a large gravimetric methane adsorption capacity (171.36 mg/g) at 100 kPa and 196 K. Thus, MOFs have led to satisfactory results at the laboratory level in the case of low-concentrated methane separation from mixtures [23,24], with no experiences being performed at greater scales. 

Therefore, most of the experimentation at lab scale and the properties’ studies are done on the original powder form, since the most-used techniques for the MOFs synthesis are solvothermal methods, which generally produce powders [25]. Industrial-scale difficulties occur as a result of pressure drops associated with powder-filled beds, high diffusional problems and low density of the materials [26,27]. In order to reduce the pressure drop through the bed, there are techniques for increasing particle size and MOF densification: mechanical, hydraulic or hot pressing, extrusion, solid or emulsion templating, and the use of a polymeric binder [28,29]. In addition, there are also other techniques currently in development, such as the sol-gel monolithic synthesis [30]. Among them, mechanical compression is an inexpensive procedure and avoids the use of additional components like polymeric binders, which may change the physical properties of MOFs [31]. However, compression pelletization could also induce amorphization as well as phase transformations, which could influence also the adsorption capacities of the MOFs [32,33].

In this way, several studies deal with the effect of mechanical compression on hydrogen adsorption for MOF-5 [34,35] and MIL-101 [36] MOFs; as well as on CO_2_ adsorption [37]. By contrast, there are fewer works related to the influence of MOFs’ densification on the methane adsorption. For example, Yuan et al. have studied the behavior of PCN-250 on the methane and nitrogen adsorption at densification pressures up to 300 MPa [38]. General pressure-effects are, added to the loss of gravimetric performance, an increase in the volumetric adsorption capacity, in addition to higher stability in a humid ambient. Typically, these adsorption studies are done at elevated gas pressures since the main objective is to increase material density and volumetric adsorption capacity for meeting gas storage challenges. In this work, the aim is the separation of methane from low-concentration streams, so adsorption studies have been conducted at low pressure (0.1 MPa). 

Therefore, the aim of this work is to study, firstly, the pressure-induced changes on the morphology and structure of three of the most common (and commercially available) MOFs, Basolite C300, Basolite F300 and Basolite A100; and, secondly, on the methane and nitrogen adsorption capacity at low pressure (0.1 MPa). The study of the adsorption capacity for methane (component to be recovered in VAM) and nitrogen (majority component in VAM) establishes a benchmark for the use of these commercial materials at industrial scale for obtaining profiting lean emissions as a novel energy source.

## 2. Materials and Methods 

Basolite C300 [Cu_3_(C_9_H_3_O_6_)_2_], Basolite F300 (C_9_H_3_FeO_6_) and Basolite A100 (C_8_H_5_AlO_5_) were manufactured by BASF and supplied by Aldrich (96% mass basis purity, Steinheim, Germany). All three materials were stored in a desiccator in order to avoid its contact with the ambient air. Particles were used in powder form, being the commercial size: Basolite C300 (16 µm, D50), Basolite F300 (5 µm) and Basolite A100 (32 µm, D50). Methane (CH_4_), nitrogen (N_2_) and helium (He), with a purity >99.995% mol, were supplied by Air Liquide (Madrid, Spain).

The pelletization method was performed using a hydraulic press (Graseby SPECAC 15.011, Orpington, UK) at compression pressures of 55.9, 111.8 and 186.3 MPa, for 30 s. Starting pressure was selected considering two considerations: ensuring the pelletization of the material to work at the actual conditions, and the lower operating limit of the hydraulic press used for this purpose. The resulting pellets were crushed and sieved in order to obtain powder (<50 µm) to perform all the successive analysis.

Breakthrough adsorption curves were obtained by flowing either CH_4_ or N_2_ (60%) diluted in He with a total flowrate of 50 mL/min, 298 K and 0.1 MPa of total pressure in a Micromeritics AutoChem II 2920 apparatus (Norcross, GA, USA) through a fixed bed of each sample (30 mg). The evolution of CH_4_, N_2_ and He signals were followed in a Pfeiffer vacuum Omnistar Prisma mass spectrometer (Pfeiffer Vacuum, Asslar, Germany). Adsorption gravimetric capacity was obtained from desorption experiments that were performed in the same apparatus flowing a He stream (20 mL/min and 0.1 MPa) with a temperature ramp of 5 K/min from 298 K to 463 K, recording also the outlet with the mass spectrometer. 

The textural characteristics of specific surface area and pore volume were estimated by N_2_ physisorption at 77 K in a Micromeritics ASAP 2020 surface area and porosity analyzer (Norcross, GA, USA). Physisorption data was processed using Brunauer–Emmett–Teller (BET), Barrett–Joyner–Halenda (BJH) and t-plot approaches for determining surface area, total mesopore volume and total micropore volume, respectively. Variations in average pore size were calculated by assuming pore cylindrical geometry. Scanning electron microscopy (SEM) images were obtained by using a JEOL 6610LV scanning electron microscope (JEOL, Yvelines, France). The samples were coated with gold prior to observation. 

The crystallographic structures of the materials were determined by powder X-ray diffraction (PXRD) using a Philips PW 1710 diffractometer (Koninklijke Philips, Amsterdam, The Netherlands), working with the Cu-K_α_line (λ = 0.154 nm) in the 2θ range of 5–85° at a scanning rate of 2°/min. Variations in the materials cell structure were verified by the Bragg law. Consequently, variations in lattice parameters of the structures were obtained through the standard equations for cubic, orthorhombic and monoclinic structures.

## 3. Results

### 3.1. Materials Characterization

**Figure 1:**
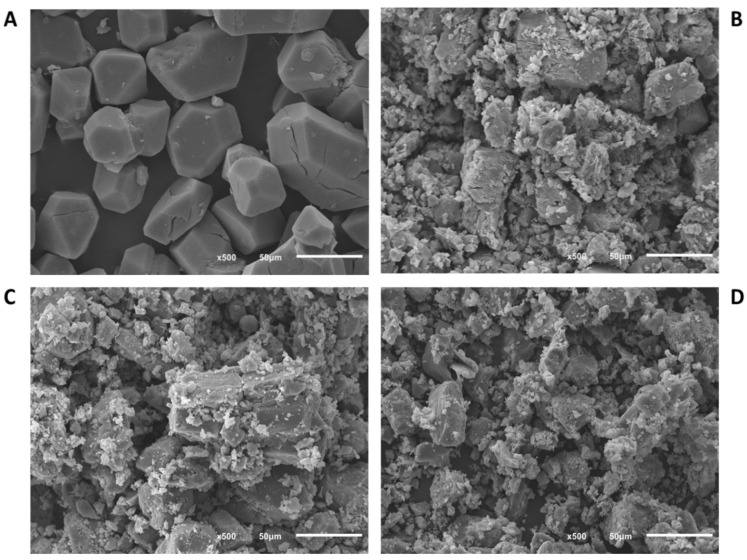
Scanning electron microscope (SEM) images of Basolite C300 (zoom in 50 µm) ((**A**): original, (**B**): 55.9 MPa, (**C**): 111.8 MPa, (**D**): 186.3 MPa).

**Figure 2:**
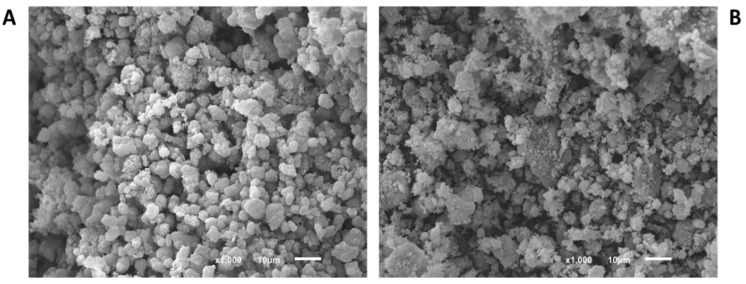

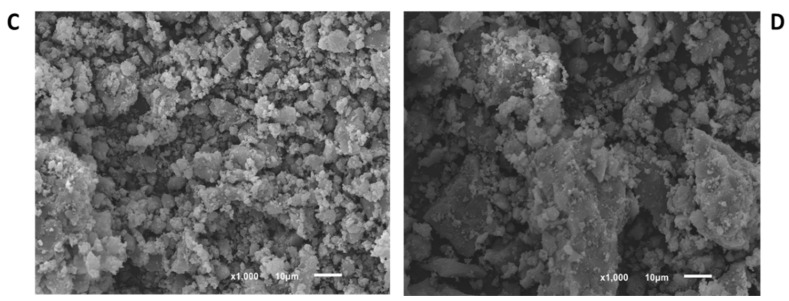
SEM images of Basolite F300 (zoom in 10 µm) ((**A**): original, (**B**): 55.9 MPa, (**C**): 111.8 MPa, (**D**): 186.3 MPa).

**Figure 3:**
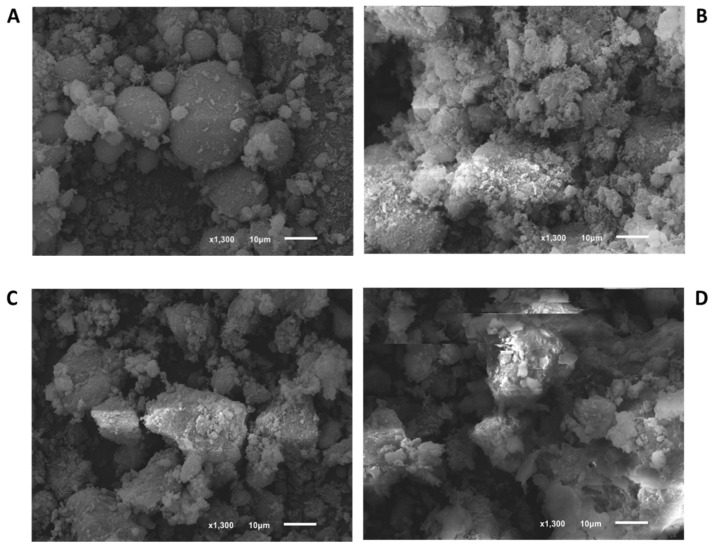
SEM images of Basolite A100 (zoom in 10 µm) ((**A**): original, (**B**): 55.9 MPa, (**C**): 111.8 MPa, (**D**): 186.3 MPa).

Figure 1, Figure 2 and Figure 3 show the SEM images of powder in the commercial form, as well as the sieved powder after the three pressure treatments. As can be observed, materials in the original form show well-defined particulate shapes, polyedric form in case of Basolite C300, and rounded shape in the case of Basolite F300 and Basolite A100. Size distribution seems to be wide for all of them, being the original size order: Basolite A100 > Basolite C300 > Basolite F300. Pressure increments lead to particle fragmentation, with the subsequent formation of irregular particle agglomerates. At the highest pressure (186.3 MPa) individual particles are practically indistinguishable, which become part of a large individual no-shaped bulk, especially for Basolite C300 and A100.

**Figure 4:**
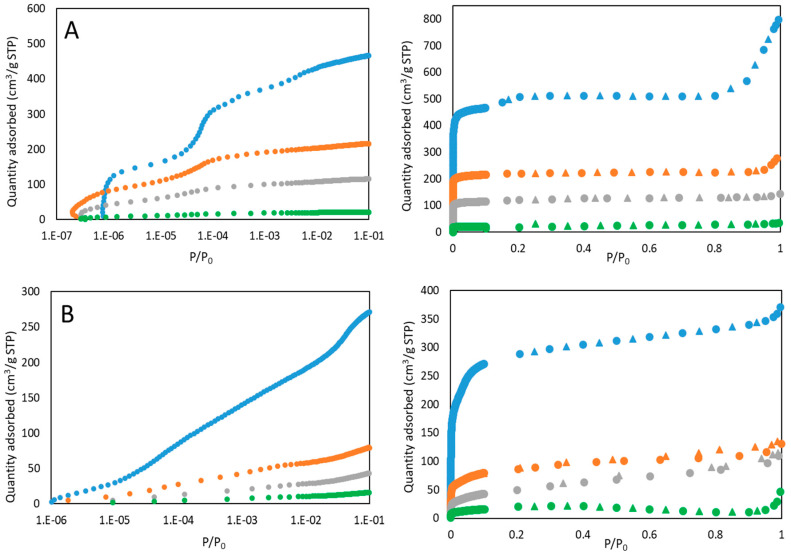

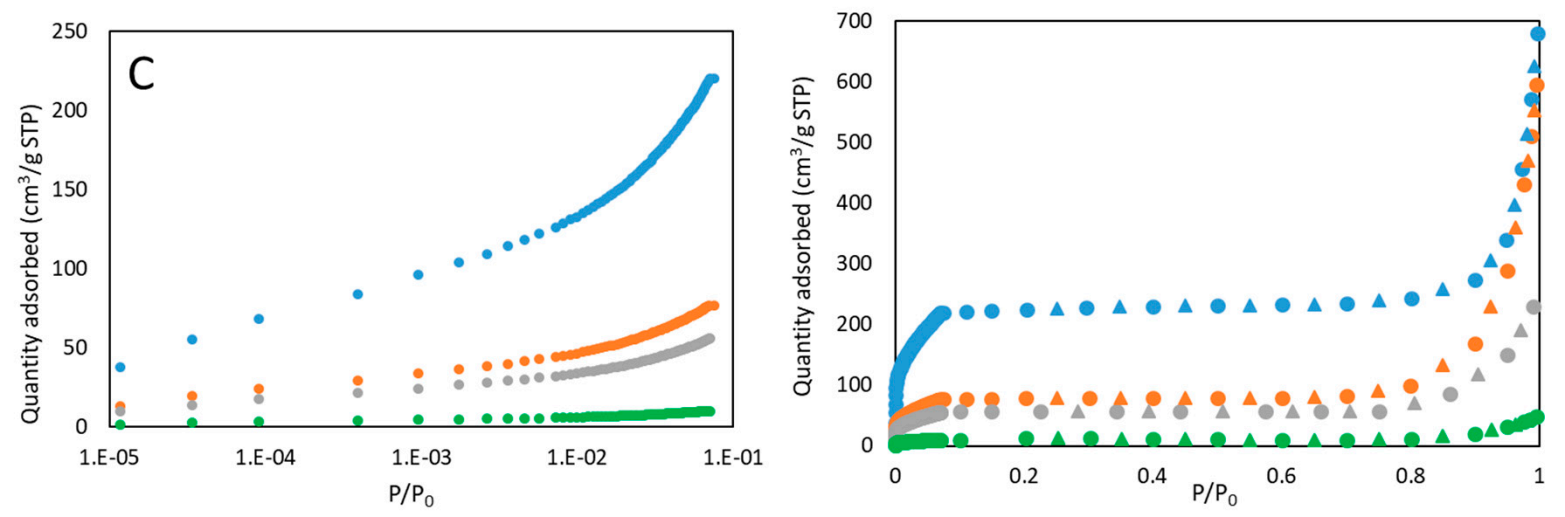
Adsorption (●) and desorption (▲) N_2_ isotherms (77 K). Basolite C300 (**A**), Basolite F300 (**B**) and Basolite A100 (**C**). Original sample (Blue), 55.9 MPa (Orange), 111.8 MPa (Grey) and 186.3 MPa (Green). The graphs on the left are zoom of the low pressure zone (up to P/P_0_ = 0.1) on logarithmic scale.

Figure 4 shows the adsorption-desorption isotherms determined by N_2_ physisorption analysis at 77 K. As shown in the figure, pristine samples exhibit a combination of type I (b) and type II isotherms, according to the International Union of Pure and Applied Chemistry IUPAC. The first zone (up to P/P_0_ = 0.8) resembles a type I (b) isotherm, with a steep elevation of the adsorbed quantity at very low pressure, and a subsequent maintenance. It is characteristic of microporous materials with wide micropores and possibly narrow mesopores [39]. The second area, up to a P/P_0_ = 1, shows a more pronounced increase of adsorbate retained, which resembles the final part of a type II isotherm. This indicates the adsorption onto macroporous or non-porous materials in multilayer disposition, which corresponds to the external phase of MOFs [40]. A combination of these two isotherms usually results in a type IV isotherm, but in this case no characteristic hysteresis is observed, and the end of the isotherms is not a plateau [41]. As the densification pressure increases, the isotherms are closer to type I (b), due to the material agglomeration and the consequent loss of external surface availability. In addition, in all the materials a marked reduction is observed in the quantity adsorbed at low P/P_0_ after pressure compression, indicative of a reduction in the total micropore volume, as it can be seen in the expanded graph (Figure 4). Micropores are clogged when particles are agglomerated with each other, in agreement with SEM images (Figure 1). The results show a significant effect of pelletization pressure on the morphology of the three MOFs (Table 1). Basolite C300 exhibits the highest BET surface loss (95.4%) at the highest pressure, although even at 55.9 MPa, the BET surface decrease reaches a value of 54.2%, in addition to 69.4% for total pore volume, which rules out the appearance of mesopores in the structure. In agreement, Casco et al. [42] have observed a great structural collapse by applying mechanical pressure (1.5 tons) to this material.

Basolite F300 presents high decreases in specific surface area (up to 93.3%) and micropore volume (96.3%), but lower in mesopore volume (up to 56.8%). The sharp BET decrease at 55.9 MPa shows the ease with which micropores collapse. However, the scarce total mesopores volume variation in the whole pressure range indicates the appearance of narrow mesopores in the structure (Table 1), as it is confirmed by the presence of some hysteresis (H4 type, according to IUPAC) at high P/P_0_ values, marked in case of 55.9 and 111.8 MPa. For this material, the appearance of two leaps in total mesopore volume value is also remarkable, one between original material and 55.9 MPa and the other between 111.8 and 186.3 MPa. This indicates that the appearance of mesopores is higher at 111.8 MPa, increasing the total mesopore volume even above of the previous applied pressure (55.9 MPa). Despite that, the total pore volume is reduced (0.15 to 0.13 cm^3^/g) in that pressure increment. This could be attributed to the formation from the voids of interparticular pore volume, as a result of the compaction.

Finally, Basolite A100 presents also high specific surface and total pore volume losses, but with a different trend than the others. The first applied pressure (55.9 MPa) provokes the highest BET surface decrease (42.1%). However, the following pressure does not affect greatly either the BET surface or the pore volume (Table 1). In agreement, Ribeiro et al. [43], after application of 62 and 125 MPa to the material, observed null relation between applied pressure and the morphological parameters, obtaining really similar results for both pressures. Finally, at the maximum pressure, a BET surface and total pore volume decrease of 94.6% and 93.3% was reached, respectively.

**Figure 5:**
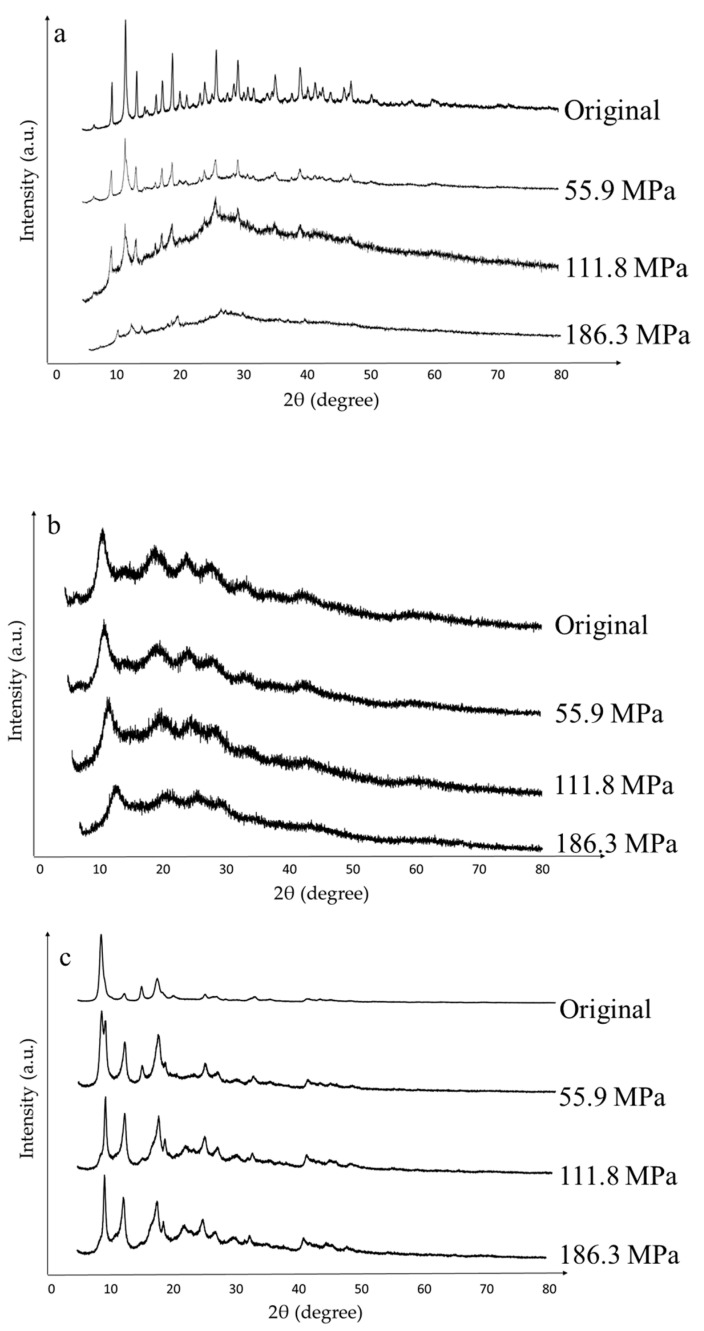
Powder X-ray diffraction (PXRD) patterns of the three materials at different applied pressures ((**a**): Basolite C300, (**b**): Basolite F300 and (**c**): Basolite A100). Applied pressures are ordered from top to bottom in increasing order (0, 55.9, 111.8 and 186.3 MPa).

Figure 5 illustrates the effect of the applied pressure on the crystallinity of both pristine and pressure-modified MOFs. The relative crystallinity is obtained by comparison of the main peak among the series of each material, assuming 100% of crystallinity for the commercial material (Table 2) [44]. In addition, peaks’ displacement along the x-axis and the appearance of new ones may mean changes in the material structure (Table 3).

The pristine Basolite C300 powder X-ray diffraction (PXRD) pattern shows the typical peaks reported for this material at 2θ = 6.7°, 9.5°, 11.65°, 13.5°, 19.3° and 26°, in addition to three little peaks at 35.5°, 38.7° and 36.43°, which indicate some CuO and Cu_2_O impurities [45,46]. After pressure is applied, the intensity of the peaks decreases progressively, indicative of crystallinity loss (Table 2). As its PXRD pattern is practically coincident with HKUST-1, a face-centered cubic structure is assumed [47], consisting of 16 copper atoms, 8 at the corners, as well as 6 at the center of the cube faces. Low-angle peaks (9.5°, 11.65° and 13.5°) present (220), (222) and (400) as Miller indices [48]. The net parameter (a) is obtained from the lattice plane of (222), Table 3. As shown, the cell volume remains practically unalterable (maximum variation of 1%), due to the structure rigidity [49]. In agreement, McKellar et al. reported variations of 2.6% for densification pressures of 3.9 GPa [50]. Likewise, the non-appearance of new crystalline peaks indicates that the cubic structure is maintained [51,52]. Therefore, the pressure effect on Basolite C300 consists of crystallinity destruction, in agreement with the BET surface area and pore volume reduction with the pressure, but remaining unaltered the cubic structure of unaltered cells. In agreement, Peng et al. [53] have studied the effect of mechanical pressure (up to 5 tons) onto HKUST-1, indicating a great micropore volume loss (N_2_ physisorption analysis), in addition to a total collapse of the crystalline structure (PXRD analysis).

For pristine Basolite F300, a characteristic peak at 2θ = 11° is observed, despite the low resolution of the pattern as a consequence of the semiamorphous nature of the material and the elevated background values due to the iron fluorescence [54]. In fact, Basolite F300 is a distorted form of crystalline MIL-100(Fe) [55], and possesses a zeolite MTN topology [56]. In this case, the semiamorphous nature of the material just allows observing an increase of the amorphous matter with the pressure (Table 2). As it is a semiamorphous material, crystallinity is slightly reduced in relative terms [57], not reflected in the BET surface, which does not depend on crystallinity and is severely affected by increased pressure. Particle agglomeration causes the collapse of micropores, as it was demonstrated in Figure 4. 

Finally, in the case of Basolite A100, this shows a structure practically coincident with MIL-53(Al) MOF, with characteristic peaks at 2θ = 8.8°, 15.25° and 17.75° [58]. The original pattern obtained is close to that of the large-pore (lp) phase of MIL-53(Al), which is coincident with an orthorhombic structure [59]. For this result, three different net parameters make up the structure and all the angles are right. Diffracting planes that match the characteristic peaks are (101), (011) and (210) [60,61]. As the applied pressure progresses, the appearance of new peaks around 2θ = 20° indicates a movement to the narrow-pore (np) phase [62,63]. In this case, structural changes are high, due to the phase transition, reaching differences up to 13.7% for total cell volume (Table 3). In fact, according to Ghoufi et al. [64], the cell shows a monoclinic structure [65] from, approximately, 53 MPa onwards. As observed, transition to the np structure has an associated reduction of the total cell volume, as well as a decrease of the *a* parameter, in conjunction with increasing trends in the rest of the parameters, including the β angle. This increase in the β angle denotes a flattening on one of its axes [62,66], being these phase changes reversible [59]. Thus, this structure is characterized by its great flexibility. Regarding crystallinity, after the initial loss at the lowest pressure, it remains practically unchanged. The first applied pressure changes the material structure to np phase, which is known for its high resistance to external pressure and flexibility [63], thus maintaining crystallinity for successive applied pressures. The same occurs at 55.9 and 111.8 MPa in the case of BET available surface and total pore volume, which are practically maintained after an abrupt decrease despite the increase of applied pressure.

### 3.2. Performance Analysis

**Figure 6:**
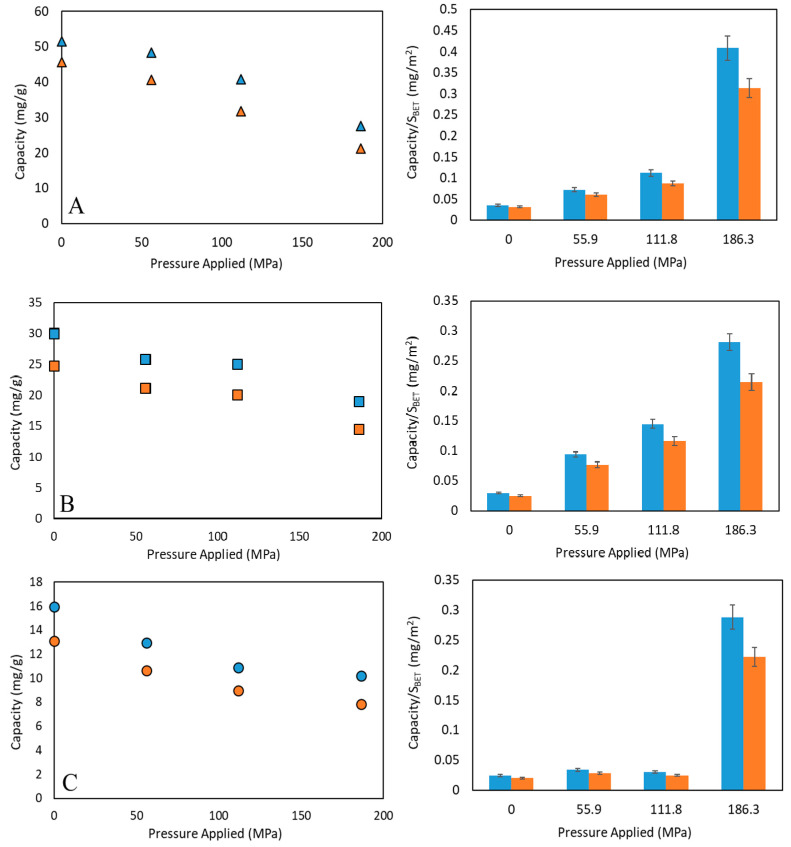
Adsorption capacity for pure methane (blue) and nitrogen (orange) for different applied pressures at 298 K and 0.1 MPa of total pressure (figures on the left), and its relation with BET specific surface area (figures on the right). Basolite C300 ((**A**), ▲), Basolite F300 ((**B**), ■) and Basolite A100 ((**C**), ●).

The gravimetric adsorption capacity of the samples was calculated from desorption analyses. Figure 6 plots adsorption capacity at different applied pressures as well as the relationship between adsorption capacity and BET specific surface area for each material. Basolite C300 shows a dramatic total decrease of its adsorption capacity with applied pressure, following a progressive trend as in the case of crystallinity and BET surface area. After the first pressure applied, some microporosity is still available, observing decreases of the adsorption capacity of 10.8% for nitrogen and 6.25% for methane. A further pressure increase will led to the total loss of adsorption capacity, BET surface and crystallinity. Additionally, the adsorption capacity/BET surface area ratio is practically linear at low applied pressures, showing certain dependence on BET surface. At the highest pressure, a sharp increase is observed, probably due to the increased role of active metal centres in the adsorption, once the crystalline structure was collapsed. 

In the case of Basolite F300, a decreasing trend of the capacity of adsorption with the applied pressure is observed, the downward trend being more pronounced at the highest pressure (loss of 41.3% for N_2_ and 36.5% for CH_4_), Figure 6B. Adsorption capacity follows a similar trend to BJH total mesopore volume (Table 1), which could be related to its originally semi-amorphous properties, in which the adsorption capacity is not drastically reduced until a certain pressure limit. The accessibility to metal adsorption sites is maintained due to the appearance of mesopores and, thus, the intracrystalline diffusivity increases. This increase in accessibility is closely related to the smooth downward trend in adsorption capacity, showing an almost linear relationship with the specific available surface. 

For Basolite A100, a sharp decrease is observed after the first applied pressure, coincident with the asymptotic trend of BET surface area to the last applied pressures (Figure 6). This may be due to the presence of pure CH_4_ and N_2_, which provokes the transition to the lp phase at ambient conditions, thus increasing the adsorption capacity by increasing the accessibility to metallic adsorption centers [67]. In fact, from adsorption capacity/BET surface ratio, a constant behavior is observed at the lowest pressures, and a sudden increase at the highest one, due to the drastic reduction of specific surface area after the transition to the lp phase which allows the metallic adsorption centers to have great relevance in the adsorption. Comparing this with other techniques, Finsy et al. [68] have studied the effect of making pellets of MIL-53(Al) using polyvinyl alcohol as a binder. They indicated a reduction of 32% in micropore volume with a pore accessibility reduction of 19% in the best of the cases, which hinders adsorption processes. In fact, it must be pointed out that the presence of a binder can affect the adsorption behavior of the material [69]. 

**Figure 7:**
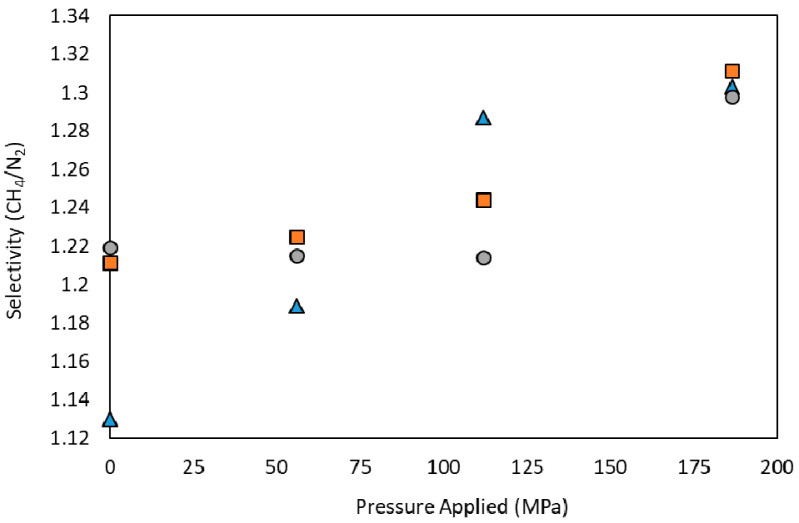
Adsorption CH_4_/N_2_ selectivity (mass basis) for each material at different applied pressures, at 298 K and 0.1 MPa of total pressure. Basolite C300 (▲), Basolite F300 (■) and Basolite A100 (●).

From Figure 6 it is observed that the relative adsorption capacity decreases are higher for N_2_ than for CH_4_, and it may be related to metal adsorption centers being available, and more selective towards CH_4_ than N_2_ [70]. Thus, after surface area and total pore volume reduction, the available active metallic centers play a more relevant role in the selective gas adsorption, especially in Basolite C300 and A100 cases. The influence of the applied pressure in the CH_4_/N_2_ selectivity (mass basis) is shown in Figure 7. The increasing slope for Basolite C300 is markedly higher than for the other materials, due to the presence of a higher percentage of metal in its structure (31.5% of copper, vs. 21.2% and 12.9% of iron and aluminum for Basolite F300 and A100, respectively). Therefore, the higher metal content in the structure, the greater the selectivity-increasing trend with applied pressure.

**Figure 8:**
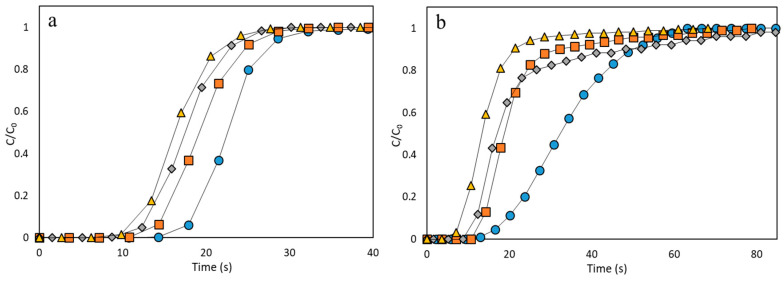

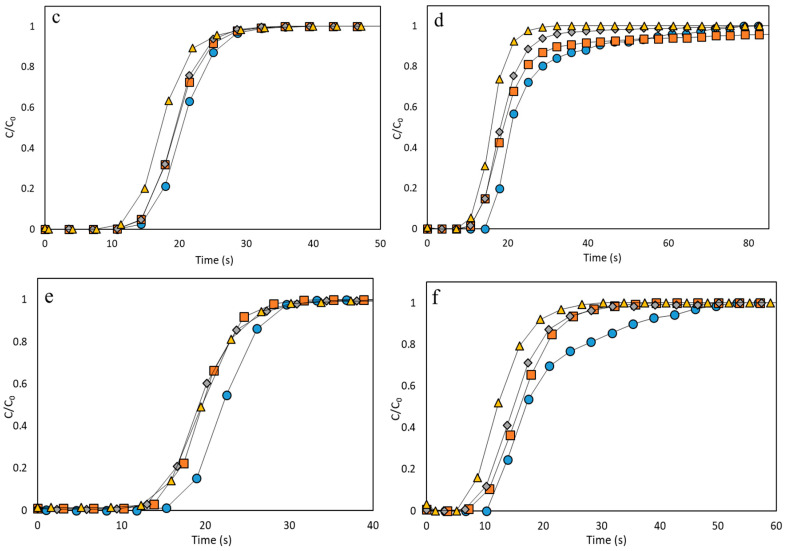
Adsorption breakthrough curves for CH_4_ and N_2_ onto the three MOFs at different applied pressures (Original: blue ●, 55.9 MPa: orange ■, 111.8 MPa: grey ♦, 186.3 MPa: yellow ▲). Basolite C300 ((**a**): methane, (**b**): nitrogen), Basolite F300 ((**c**): methane, (**d**): nitrogen) and Basolite A100 ((**e**): methane, (**f**): nitrogen). Black lines are used to guide the view.

Breakthrough adsorption curves for CH_4_ and N_2_ in a fixed bed are shown in Figure 8. In general, for all the samples, breakthrough times (hence, adsorption capacity) are higher for CH_4_ than for N_2_, being attributed to the presence of metallic active adsorption sites and the difference in polarizability of both molecules [70]. 

In the case of Basolite C300, there is a slight difference in the slope between the original material and the others, most obvious in N_2_ case. As N_2_ molecular size is lower than CH_4_ (3.65 and 3.82 Å, respectively), this molecule may penetrate in narrower pores than CH_4_. Likewise, a decrease in the Knudsen diffusion coefficient led to more inclined curves [71,72]. The Knudsen diffusion coefficient (D_K_) depends on the pore diameter (d_p_), since the other parameters are constant for all the experiments. Variations in the Knudsen diffusion coefficient affects directly the breakthrough curve, since it influences adsorbate mass transfer kinetics within the microporous adsorbent.

The reduction in total available specific surface, especially in the micropores zone (low P/P_0_), indicates that these narrower pores have been totally collapsed by compression (Figure 4). This collapse is common in MOFs when pressure is applied, due to their extraordinary initial porosity [73]. This provokes the following applied pressures to present less-inclined breakthrough curve slopes, but also having less adsorptive capacity, as evidenced by the x-axis order of their breakthrough times (Figure 8). Breakthrough times follow, approximately, the same trend as BET surface area. 

In the case of Basolite F300, all the samples, except the original one, show the same slope for breakthrough curves, but in this case the difference is lower than in C300 case. The original sample presents a more inclined breakthrough curve for both adsorbates, which indicates a lower Knudsen diffusion coefficient. Applied pressure modified the pore structure, plugging the micropores, but without reducing greatly the total pore volume by the appearance of mesopores that facilitate the penetration, so the differences in accessibility are softer (Figure 4). The high resemblance between the 55.9 and 111.8 MPa curves (Figure 8) is remarkable and can be related to the no-clear total mesopore volume dependence on pressure (Table 1). The appearance of mesopores in the structure enhance the intracrystalline diffusivity [74], which may be the dominant factor in this case, since the crystallinity is not great affected by mechanical pressure. Dhakshinamoorthy et al. have studied the high relevance of the intracrystalline diffusivity in this material, applied to the case of an oxidation reaction [75]. 

Finally, in the case of Basolite A100, despite the change from orthorhombic to monoclinic structure and the total cell volume reduction, the presence of pure CH_4_ and N_2_ causes the return to the lp phase at ambient conditions, for which the penetration is easier, obtaining a steep curve for all cases due to the structure flexibility [76]. As is also observed, the breakthrough curves of the original material present more resistance than the others, especially for N_2_. Despite the return to lp phase, the agglomeration provoked a certain irreversible reduction of micropore volume, which increases the Knudsen diffusion coefficient since the average available pore size is higher (Table 1). It is remarkable that differences in CH_4_ breakthrough times follow almost the same trend as crystallinity, whereas in the N_2_ case, the trend is similar to specific surface or total pore volume.

## 4. Conclusions

Structural and morphological transformations of three MOFs (Basolite C300, Basolite F300 and Basolite A100), as well as CH_4_ and N_2_ uptakes variation, were studied after appliance of mechanical pressure to the materials. Basolite C300, a rigid crystalline material, experimented a dramatic and progressive loss of crystallinity, as well as surface area and pore volume, which implies lower adsorption capacity due to its characteristic pores collapse. In the case of Basolite F300, a semiamorphous material, this experienced also a high decrease of surface area and micropore collapse due to agglomeration, but keeping total pore volume due to the appearance of mesopores in the structure. This transformation implies an increase of intracrystalline diffusivity and, then, lower adsorption capacity losses. For Basolite A100, a flexible crystalline MOF, a transformation is observed from orthorhombic disposition to monoclinic structure from 55.9 MPa onwards, in addition to high permanent losses of microporosity due to agglomeration. This structure change is reversible, returning to the lp phase in presence of CH_4_ and N_2_ at ambient conditions. This fact increases the accessibility to metallic active centers and an asymptotic decrease of the adsorption capacity is observed. Additionally, the key role of metal active sites in the CH_4_/N_2_ selectivity was pointed out. In fact, an increased selectivity for the three MOFs was observed with the applied pressure, decreasing this positive effect in the order: Basolite C300 (Cu, 31.5%) > Basolite F300 (Fe, 21.2%) > Basolite A100 (Al, 12.9%). However, the total gravimetric adsorption capacity has experienced high losses for all of them. Despite that, Basolite C300 stands out above the other two. It has greater adsorption capacity and also a higher metallic content in its structure. In addition, it is able to retain 94% of its adsorption capacity when applying a pressure of 55.9 MPa, enough to increase its particle size and be able to operate in real adsorption stages. 

## Figures and Tables

**Table 1:** Variations of Brunauer–Emmett–Teller (BET) surface area, Barrett–Joyner–Halenda (BJH) total mesopore volume, *t*-plot total micropore volume and average pore size with applied pressure.

Material	Applied Pressure (MPa)	BET Specific Surface Area (m^2^/g)	BJH Mesopore Volume (cm^3^/g)	*t*-Plot Micropore Volume (cm^3^/g)	Average Pore Size (Å)
C300	0	1466.8	0.53	0.71	33.7
	55.9	671.2	0.09	0.29	22.9
	111.8	364.8	0.07	0.14	23.2
	186.3	67.6	0.06	0.02	47.3
F300	0	1015.4	0.15	0.27	16.5
	55.9	276.2	0.09	0.06	22.3
	111.8	173.4	0.11	0.02	28.8
	186.3	67.8	0.06	0.01	41.2
A100	0	655.9	0.77	0.28	64.0
	55.9	380.2	0.57	0.06	65.8
	111.8	362.1	0.54	0.05	65.1
	186.3	35.4	0.06	0.01	70.1

**Table 2:** Relative crystallinity losses associated with applied pressure (referred to the original material).

Material	Pressure (MPa)	Crystallinity Loss (%)
C300	0	0
	55.9	19.51
	111.8	62.07
	186.3	69.93
F300	0	0
	55.9	0
	111.8	4.31
	186.3	14.08
A100	0	0
	55.9	69.35
	111.8	68.52
	186.3	68.38

**Table 3:** Cell total volume and lattice parameters for each structure depending on applied pressure.

Material	Pressure (MPa)	a (Å)	b (Å)	c (Å)	α (°)	β (°)	γ (°)	Volume (Å^3^)
C300	0	25.9	25.9	25.9	90	90	90	17,452.2
	55.9	26.2	26.2	26.2	90	90	90	17,992.8
	111.8	26.2	26.2	26.2	90	90	90	17,992.8
	186.3	26.1	26.1	26.1	90	90	90	17,901.2
A100	0	16.1	6.56	13.2	90	90	90	1397.4
	55.9	6.56	14.3	14.8	90	105	90	1351.7
	111.8	6.43	12.9	16.2	90	108	90	1280.5
	186.3	5.83	13.7	16.1	90	110	90	1205.4
